# Retrieval-augmented clinical decision support for structured hip-joint disease assessment

**DOI:** 10.3389/fmed.2026.1890014

**Published:** 2026-07-17

**Authors:** Qing-Yuan Long, Guan-Yu Wang, Wu-Long Yang

**Affiliations:** The Second Affiliated Hospital of Guizhou Medical University, Kaili, China

**Keywords:** clinical decision support, hip disease, large language model, retrieval-augmented generation, retrospective validation

## Abstract

**Background:**

Hip-joint disease assessment requires integration of symptoms, imaging findings, staging criteria, and management context. Clinician-facing decision-support systems may help structure this reasoning but require defined clinical evaluation.

**Methods:**

We conducted a retrospective, case-based validation of a hip-joint decision-support system integrating a local knowledge base, retrieval-augmented generation, and a multi-agent reasoning workflow. Seventy-four cases across five disease categories, stratified as easy, moderate, or complex, were evaluated. Validation diagnosis labels were established by an independent expert panel. Fifteen physicians (5 consultants, 10 residents/fellows) generated 1,110 evaluations. Endpoints included diagnostic accuracy, physician-rated clinical domains, confidence, decision time, and perceived acceptability.

**Results:**

Overall accuracy was 85.1% (95% CI, 76.8–93.4) for Hip-Agent, 94.6% (92.3–96.9) for consultants, and 73.5% (70.3–76.7) for residents/fellows. Hip-Agent accuracy was 94.4 and 92.0% in easy and moderate cases but 46.2% in complex cases. Inter-observer reliability for clinical-domain ratings was good to excellent (ICC 0.79–0.84). Perceived acceptability of the output format was high [mean 4.60 (0.49)].

**Conclusion:**

The system demonstrated feasible retrospective performance for hip-joint case evaluation, with performance varying by complexity. Low accuracy in complex cases warrants caution. This study did not evaluate human-AI interaction; prospective workflow evaluation is needed to assess whether the system supports clinicians’ judgment in practice.

## 1 Introduction

Hip-joint diseases comprise a clinically heterogeneous group of disorders that frequently present with overlapping pain patterns, gait disturbance, and functional limitation (1–3). Hip pain may arise from degenerative cartilage disease, ischemic injury to the femoral head, developmental abnormalities of acetabular coverage, traumatic fracture, or intra-articular pathology involving the labrum and surrounding structures (4, 5). Conditions such as osteoarthritis, avascular necrosis of the femoral head, developmental dysplasia of the hip, hip fracture, and femoroacetabular impingement or labral-related disorders often require integration of clinical history, physical examination, radiography, magnetic resonance imaging, staging or classification criteria, and treatment context (6–8). In routine orthopedic practice, the diagnostic question is therefore rarely limited to naming a disease entity; it commonly extends to judging severity, recognizing imaging features, identifying the appropriate classification framework, and linking the assessment to conservative treatment, surveillance, or surgical planning (1, 3, 5, 6). Degenerative hip osteoarthritis may share symptoms with early avascular necrosis, dysplasia-related overload, referred pain, or labral pathology, while radiographic changes can be subtle, discordant with symptoms, or dependent on disease stage (3, 5, 6). Avascular necrosis requires attention to collapse, subchondral fracture, and femoral-head morphology; dysplasia requires assessment of acetabular coverage and associated intra-articular injury; traumatic fracture requires timely recognition and management within an older or medically vulnerable population (4, 5, 7). Guidelines and consensus statements help standardize management, but their application still requires case-level interpretation across symptoms, imaging, patient factors, and treatment options (2, 3, 6–8). Less experienced physicians may therefore benefit from structured support that makes disease-specific reasoning explicit, particularly when cases involve atypical presentations, mixed imaging findings, or the need to connect diagnostic classification with management planning.

Artificial intelligence has been increasingly explored in orthopedic surgery, musculoskeletal imaging, and clinical decision support, but many systems remain organized around individual tasks or single-output prediction models, such as image classification, segmentation, fracture detection, implant planning, or prediction of a single outcome (9–13). These approaches can be useful within a defined workflow, yet they cannot support integrated differential diagnosis, staging or classification, treatment planning, and further-examination decisions within a single clinician-facing assessment. A clinically useful support system for hip disease should be able to synthesize narrative clinical information with imaging descriptions, retrieve relevant disease knowledge, organize differential diagnosis, support staging or classification, and provide treatment-support and further-examination suggestions. Conventional clinical decision-support systems also show that successful implementation depends not only on algorithmic performance but on workflow fit, transparency, human oversight, and the ability to present information in a form that clinicians can interpret (9, 14). Beyond their technical role, RAG and structured multi-agent workflows can support knowledge-grounded, reviewable clinical reasoning by linking generated recommendations to explicit evidence sources and clinically defined reasoning modules; this design also facilitates explainability and human oversight (15, 16).

Clinician-facing artificial intelligence systems require evaluation beyond technical feasibility. Early-stage decision-support studies should describe the clinical setting, intended users, human role, evaluation endpoints, and interaction with clinical workflow transparently, even when the study is retrospective rather than a prospective intervention (17). Reporting extensions for artificial intelligence trials and prediction models similarly emphasize clear descriptions of the AI intervention, data sources, intended use, performance evaluation, and human-AI interaction, while avoiding unsupported generalization beyond the tested setting (18–20). For a hip-joint decision-support system, retrospective validation can provide an initial assessment of diagnostic performance, performance variation across case difficulty and disease categories, physician-rated clinical reasoning domains, decision dynamics, and clinician-perceived usability. Such evaluation does not establish prospective patient benefit, but it can define whether the system produces clinically interpretable outputs and where further development or external validation is needed. For the present system, multifaceted validation is particularly necessary because Hip-Agent is not a single-output diagnostic prediction model but a clinician-facing system that structures diagnosis, staging/classification, treatment support, and further-examination suggestions for physician review. Accordingly, evaluation should extend beyond diagnostic accuracy to encompass the clinical interpretability of outputs, the validity of disease-specific reasoning structures, reliability, and perceived acceptability.

The objective of this study was to develop and retrospectively validate a clinician-facing decision-support system for hip-joint diseases that integrates a local hip-joint medical knowledge base, retrieval-augmented generation, and a structured multi-agent reasoning workflow. The system was designed to support diagnosis, staging or classification, treatment-support recommendations, and further examination suggestions for physician review. We acknowledge that the present study constitutes a retrospective, case-based validation comparing the standalone outputs of the system with physician assessments, rather than a prospective evaluation of clinical decision support in an interactive workflow. The retrospective validation assessed diagnostic performance compared with physician evaluator groups, performance across case difficulty and disease categories, physician-rated clinical domains, and secondary measures including confidence, decision time, and clinician-perceived usability. By focusing on a clinically heterogeneous hip-joint validation set, the study aimed to evaluate the diagnostic performance, clinical reasoning characteristics, and workflow-related measures of a retrieval-augmented decision-support approach for orthopedic assessment.

## 2 Methods

### 2.1 Study design

This retrospective, case-based clinical validation study evaluated a clinician-facing decision-support system for hip-joint diseases. The study was designed to assess the system as a support tool for structured clinical reasoning, without replacing physician judgement. Each retrospective case was treated as an independent clinical scenario for system inference and physician evaluation, allowing case-level comparison between model-generated decision-support outputs and clinician assessments. The study was approved by the institutional review board of the Ethics Committee of the Second Affiliated Hospital of Guizhou Medical University (2025-EthicalReview-233), with a waiver of informed consent for retrospective data use.

The evaluated system integrated a local hip-joint medical knowledge base with retrieval-augmented generation and structured multi-agent/model reasoning. The system was designed to accommodate narrative history, physical examination findings, radiographic or magnetic resonance imaging findings, and ancillary test information. Retrieved evidence was used to guide the reasoning process and to support outputs for diagnosis, disease staging or classification, treatment-support recommendations, and further examination suggestions. The system workflow and reasoning framework are described in the following Methods sections.

The validation framework compared system outputs and physician evaluations across diagnostic and clinical assessment endpoints. Diagnostic evaluation focused on case-level diagnostic assessment, whereas clinical assessment endpoints captured physician-rated reasoning domains and secondary measures including confidence, decision time, and clinician-perceived usability. These measures were used to characterize the decision-support process within a retrospective validation setting rather than to evaluate patient outcomes.

### 2.2 Case source and validation set

Retrospective hip-joint cases were obtained from the Second Affiliated Hospital of Guizhou Medical University. Eligible cases were retrospectively screened from electronic medical records between January 2023 and December 2024 and selected to represent the five target hip-joint disease categories and predefined difficulty strata. Inclusion criteria required: (1) a confirmed hip-joint disease diagnosis among the five target categories, (2) availability of complete clinical records including history, physical examination, and imaging reports, and (3) sufficient follow-up documentation to confirm the final diagnosis. Exclusion criteria included: (1) concurrent hip-joint pathologies spanning more than one primary disease category, (2) incomplete imaging or clinical data, and (3) prior surgical intervention for the index condition that substantially altered the diagnostic presentation. The validation set comprised 74 cases across five disease categories: osteoarthritis (*n* = 26), developmental dysplasia of the hip (*n* = 22), avascular necrosis of the femoral head (*n* = 15), fracture (*n* = 8), and labral tear (*n* = 3). Together, these categories span common degenerative, ischemic, developmental, traumatic, and intra-articular hip disorders encountered in orthopedic assessment. The validation diagnosis label for each case was established through expert consensus by a panel of three senior orthopedic consultants (each with >15 years of clinical experience in hip-joint disorders) who were independent from the evaluation panel. The consensus process involved independent review of all available clinical information—including history, physical examination, imaging studies, surgical findings where applicable, and follow-up records—followed by adjudication. When initial assessments disagreed, the panel discussed the case and reached a consensus diagnosis. This reference standard was established before the system evaluation.

Cases were stratified into easy, moderate, and complex categories for validation-level analysis. Case difficulty was assigned by the same independent expert panel that established the validation diagnosis labels, and assignment was completed before the system evaluation. The difficulty criteria were predefined as follows: easy cases were defined as those with a single, typical presentation consistent with classical diagnostic criteria and unambiguous imaging findings; moderate cases involved either atypical symptom patterns, mixed imaging features requiring differential consideration, or the need for staging/classification assessment; complex cases featured overlapping clinical or imaging features suggesting multiple differential diagnoses, discordant clinical and imaging findings, or presentation requiring integration of multiple classification systems. Disagreements in difficulty assignment were resolved through panel discussion and majority vote. The difficulty strata included 36 easy cases, 25 moderate cases, and 13 complex cases. This structure allowed the validation to examine case-based diagnostic assessment across both routine presentations and more challenging clinical scenarios while preserving the retrospective, case-level study design.

The physician evaluation panel included 15 evaluators, comprising 5 consultants and 10 residents/fellows. In this manuscript, “consultants” refers to senior attending orthopedic surgeons specializing in hip-joint disease management, each with more than 15 years of independent clinical experience after specialist training; residents and fellows were orthopedic trainees at the postgraduate year 3 to 6 level. All evaluators completed a standardized orientation session covering the evaluation protocol, rating-scale anchors, and output format before case assessment. Each case was evaluated by all physicians, yielding 1,110 physician-case evaluations. The validation set was summarized by disease category, difficulty stratum, evaluator seniority, and evaluation-level structure, as shown in Table 1.

**Table 1:** Validation set and evaluator composition.

Characteristic	Value
Cases	74
Physician evaluators	15
Physician-case evaluations	1,110
Consultants	5
Residents/fellows	10
Osteoarthritis	26
Developmental dysplasia of the hip	22
Avascular necrosis of the femoral head	15
Fracture	8
Labral tear	3
Easy cases	36
Moderate cases	25
Complex cases	13

Disease categories and difficulty strata are reported as case counts. Each case was evaluated by 15 physicians.

### 2.3 System architecture

**Figure 1:**
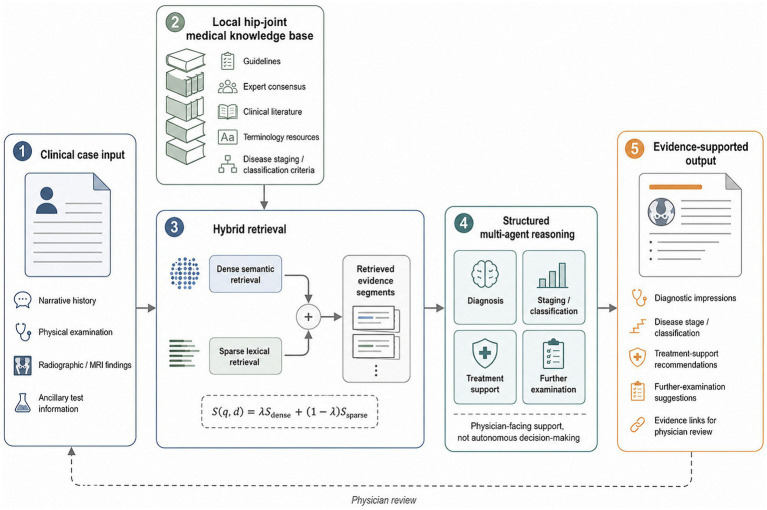
Workflow of the hip-joint clinical decision-support system. Clinical case information and curated hip-joint knowledge sources are processed through a hybrid retrieval layer that combines semantic and lexical matching. Retrieved evidence is incorporated into a structured multi-agent reasoning workflow to generate evidence-supported diagnostic, staging or classification, treatment-support, and further-examination outputs for physician review.

The decision-support system was organized as a layered clinician-facing architecture for hip-joint disease assessment. The clinical input layer accepted narrative or structured information from a case scenario, including symptoms, physical examination findings, imaging descriptions, and ancillary test information when present. These inputs were passed to a local medical knowledge base and retrieval layer before model-based synthesis, so that the generated output was linked to domain-specific orthopedic knowledge rather than produced from the language model alone. The overall workflow is summarized in Figure 1.

The functional architecture comprised five layers: clinical case input, local hip-joint medical knowledge base, hybrid retrieval, structured multi-agent reasoning, and evidence-supported output. The knowledge base layer stored curated hip-joint disease content and terminology resources. The retrieval layer combined dense semantic retrieval with sparse lexical retrieval to identify clinically relevant evidence segments. The reasoning layer then synthesized the patient-level input and retrieved evidence into structured decision-support outputs. The output layer returned clinically interpretable content for physician review, including diagnostic impressions, staging or classification support, treatment-support recommendations, and suggestions for further examination.

The system was implemented on a workflow platform, with FastGPT used as an implementation environment for knowledge-base management, retrieval orchestration, and model workflow assembly. The supporting data infrastructure was configured to store case-level records, maintain vector indexes, provide relational storage redundancy, and generate semantic representations of knowledge segments and clinical queries (MongoDB, Milvus, PostgreSQL, and Qwen-embedding-4). These components were used to support storage, retrieval, and workflow consistency within the validation system; they were not evaluated as independent clinical interventions.

### 2.4 Knowledge base construction

The local knowledge base was constructed to support hip-joint diagnosis, staging or classification, treatment-support reasoning, and further-examination suggestions. Source categories included orthopedic guidelines, expert consensus documents, hip-joint disease literature, textbook-like disease knowledge, and terminology resources relevant to orthopedic and imaging assessment. The final knowledge base comprised 47 clinical guidelines, 23 expert consensus documents, 156 peer-reviewed articles, 8 textbook-derived disease knowledge chapters, and 5 terminology resources. Source selection was performed by two orthopedic consultants based on clinical relevance, methodological quality, and temporal currency (sources published within the preceding 10 years prioritized, with landmark older references retained); inclusion required agreement between both selectors, with discrepancies resolved by a third senior consultant. The indexed knowledge base contained 2,847 discrete knowledge segments after segmentation and cleaning. A detailed source composition is provided in Supplementary Table S1.

Knowledge-base curation was guided by clinical relevance, source authority, temporal relevance, and applicability to hip-joint diagnostic and treatment-support tasks. Candidate material was organized around clinical presentations, imaging signs, disease definitions, staging or classification criteria, and management concepts. The purpose of curation was to provide a domain-specific evidence context for model generation, while avoiding any assumption that the knowledge base was exhaustive or continuously updated.

Before indexing, textual material was cleaned to reduce formatting noise and normalize terminology. Documents were segmented into knowledge units intended to balance contextual integrity with retrieval granularity: segments needed to retain enough clinical context for interpretation while remaining sufficiently focused for case-level matching. Terminology normalization and synonym handling were applied to hip symptoms, physical findings, imaging signs, diagnostic labels, staging or classification terms, and treatment concepts. This step was intended to reduce retrieval mismatch caused by variant expressions such as hip pain, groin pain, joint-space narrowing, femoral-head collapse, acetabular dysplasia, or avascular necrosis.

### 2.5 Retrieval-augmented clinical inference

For each case, the clinical input was transformed into a retrieval query that combined the salient elements of the scenario, including symptoms, examination findings, imaging descriptions, suspected diagnoses, and available ancillary information. The indexed knowledge base was searched using both dense semantic retrieval and sparse lexical retrieval. Dense retrieval used embedding-based similarity to identify semantically related knowledge segments, whereas sparse retrieval used lexical matching, such as BM25, to retain sensitivity to disease names, staging terms, imaging signs, and other specific clinical expressions (21). Hybrid retrieval was represented as a weighted combination of dense and sparse retrieval scores as shown in Equation 1:


$$S(q,d)=\lambda S_{\text{dense}}(q,d)+(1-\lambda )S_{\text{sparse}}(q,d)$$ (1)

where q denotes the clinical query, d denotes a candidate knowledge segment, $S_{\text{dense}}(q,d)$ is the embedding-based semantic similarity score, $S_{\text{sparse}}(q,d)$ is the sparse lexical retrieval score, and λ is the weighting coefficient balancing semantic and lexical retrieval. The retrieval parameters were fixed before validation: λ=0.6 , top-k = 8 evidence segments per query, embedding dimension = 1,024, and similarity threshold = 0.65. These parameters were determined using a 15-case development set that was not included in the validation cohort.

The selected evidence set was defined by Top-k retrieval from the indexed knowledge base as shown in Equation 2:


$$D_{k}(q)={\text{TopK}}_{d\in D}S(q,d)$$ (2)

where D denotes the indexed knowledge base and $D_{k}(q)$ denotes the set of retrieved evidence segments selected for the query. The retrieved evidence was supplied to the generation workflow to ground diagnostic reasoning, staging or classification support, treatment-support recommendations, and further-examination suggestions, consistent with retrieval-augmented generation methods (22–24). This retrieval-augmented design was intended to constrain generation toward relevant clinical evidence and mitigate unsupported generation, while retaining physician review as the final interpretive step.

### 2.6 Agent-based decision workflow

**Figure 2:**
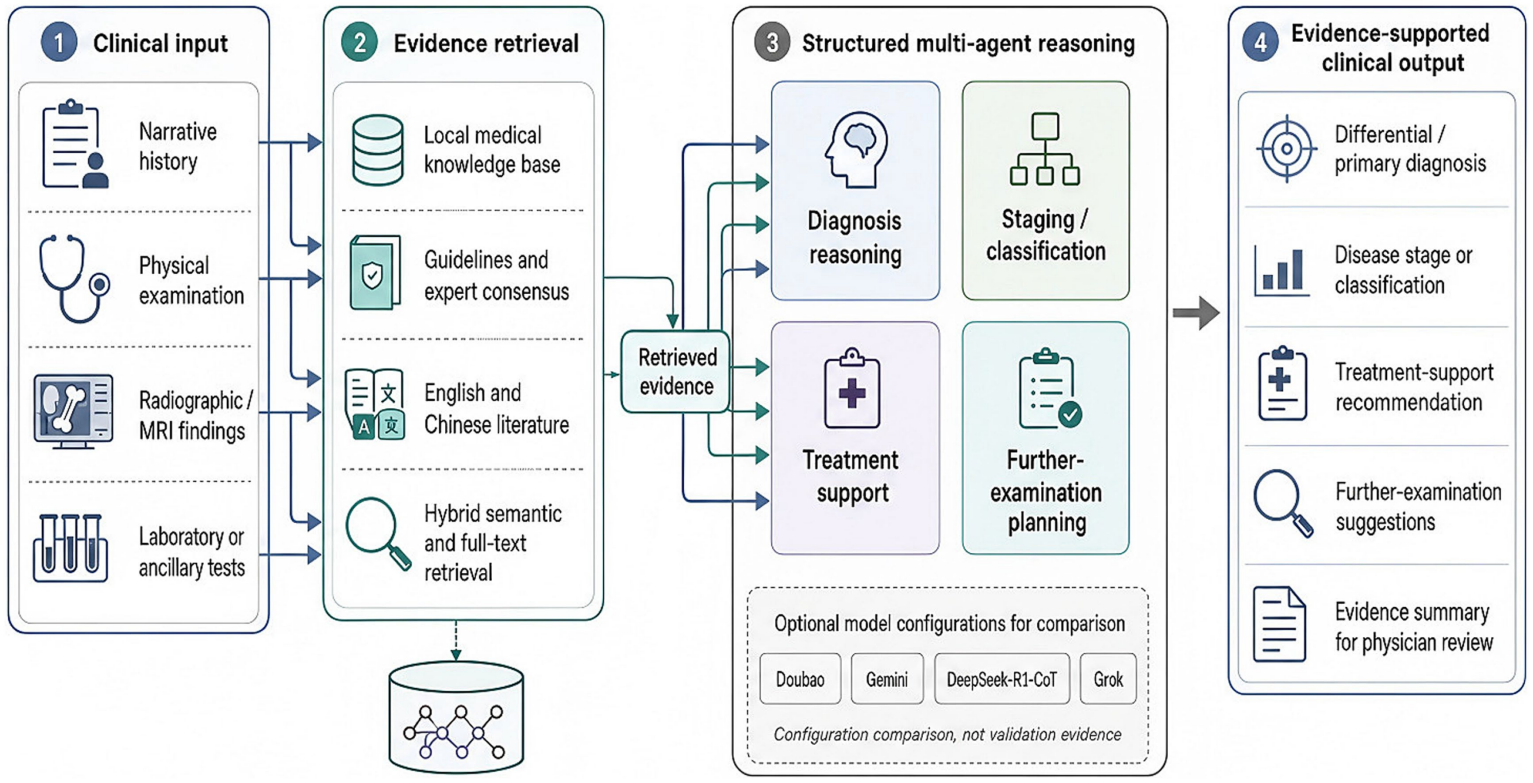
Clinical reasoning framework of the decision-support system. Clinical input and retrieved evidence are organized into task-specific reasoning modules for diagnosis, staging or classification, treatment support, and further-examination planning. Optional model configurations are shown as comparison settings rather than validation evidence. Outputs are presented as evidence-supported clinical information for physician review.

The agent-based workflow organized model outputs into clinically recognizable tasks rather than exposing hidden reasoning traces. The primary validation used DeepSeek-R1 (version 2025-01-20, accessed via API) as the generation model. Generation parameters were fixed across all validation cases: temperature = 0.3, top-*p* = 0.9, and maximum output length = 4,096 tokens. No web browsing or external retrieval beyond the local knowledge base was enabled during validation. The workflow comprised four sequential reasoning modules: (1) the diagnostic reasoning agent received clinical input and retrieved evidence, and was prompted to generate a structured differential diagnosis with the primary diagnosis, supporting evidence from retrieved segments, and alternative considerations; (2) the staging/classification agent applied disease-specific criteria (e.g., ARCO staging for avascular necrosis, Tönnis grading for dysplasia, Kellgren-Lawrence grading for osteoarthritis) to the clinical and imaging information; (3) the treatment recommendation agent generated management suggestions aligned with retrieved guideline evidence, including conservative and surgical options where applicable; and (4) the further examination suggestion agent identified additional imaging, laboratory, or specialist consultation needs. Each agent’s output was structured with explicit evidence citations from the retrieved segments, and the final consolidated output was presented as a single evidence-supported report for physician review. The workflow also allowed optional comparison across model configurations, including Doubao, Gemini, and Grok, as alternative reasoning settings rather than as independent validation evidence. This task-decomposed clinical reasoning framework is shown in Figure 2.

The generation step can be summarized as shown in Equation 3:


$$\begin{array}{c} \hat{y}=G_{\theta }(x,D_{k}(q),p) \end{array}$$ (3)

where x denotes the clinical input, $D_{k}(q)$ denotes the retrieved evidence set for the corresponding query, p denotes the structured clinical prompt, $G_{\theta }$ denotes the generation model, and $\hat{y}$ denotes the generated decision-support output.

The structured clinical prompt was designed to organize the output into differential diagnosis or primary diagnosis, disease staging or classification when applicable, treatment-support recommendations, and further-examination suggestions. An illustrative de-identified case vignette showing the structured output domains is provided in Supplementary Figure 1. The user-facing output emphasized structured clinical synthesis and organized fields rather than unstructured internal model traces. The final output was therefore positioned as evidence-supported information for physician review rather than as an autonomous clinical decision.

### 2.7 Evaluation procedure

The retrospective validation was conducted at the level of physician-case evaluation, with the human role and clinical evaluation endpoints specified in line with early-stage AI decision-support reporting principles (17). The validation set comprised 74 hip-joint cases spanning osteoarthritis, developmental dysplasia of the hip, avascular necrosis of the femoral head, fracture, and labral tear, with cases stratified into easy, moderate, and complex categories for validation-level analysis. Fifteen physicians participated in the evaluation, including 5 consultants (each with >15 years of orthopedic experience) and 10 residents/fellows (postgraduate years 3–6). Before evaluation, all participants completed a standardized orientation session covering the case-review protocol, scoring anchors, confidence reporting, decision-time recording, and the procedure for reviewing de-identified system outputs. Because each case was evaluated by each participating physician, the validation generated 1,110 physician-case evaluations.

For system inference and physician evaluation, each case was converted into a standardized de-identified case summary derived from the electronic medical record. The case summary included the clinical history, relevant symptoms, physical examination findings, radiology-report-derived imaging descriptions, and ancillary test information when available. Raw imaging files were not directly provided to the Hip-Agent or to physician evaluators during the validation task; instead, imaging information was presented as standardized textual descriptions based on radiographic or MRI reports. The same case-summary information was used for Hip-Agent inference and for physician evaluation. To avoid answer leakage, diagnostic labels, final diagnoses, radiology impressions explicitly naming the final diagnosis, treatment decisions, operative conclusions, and follow-up diagnostic conclusions were removed from the case summaries before evaluation.

For each case, the decision-support system generated structured outputs, and physician assessments were recorded within the retrospective validation framework. These outputs and assessments were subsequently compared using predefined diagnostic and clinical evaluation endpoints. For the decision-support system, binary diagnostic accuracy was calculated at the case level. For physicians, binary diagnostic accuracy was calculated at the physician-case evaluation level, with each physician assessment treated as one evaluation record.

Clinical reasoning quality was assessed using three physician-rated domains: diagnostic precision, radiologic feature recognition, and management logic. Each domain was scored on a structured 5-point Likert scale with prespecified clinical anchors developed for this study; these scales were used as descriptive evaluation measures rather than validated psychometric instruments. The scale anchors were: 1 = poor (major errors or omissions), 2 = marginal (significant limitations), 3 = acceptable (minor limitations), 4 = good (competent with minimal limitations), 5 = excellent (comprehensive and accurate). During each physician-case evaluation, the evaluator independently assessed the case and generated a diagnostic conclusion, after which they reviewed the de-identified Hip-Agent output in a standardized format and rated the output quality across the three domains. Thus, the 1–5 clinical-domain scores represent evaluator-level assessments of the system’s output quality, and are reported descriptively by evaluator seniority group. Evaluators were blinded to the source of the output when rating system-generated assessments. Inter-observer reliability was assessed using a two-way random-effects intraclass correlation coefficient (ICC) model for absolute agreement among the 15 evaluators across all rated cases, yielding ICCs of 0.82 (95% CI, 0.76–0.87) for diagnostic precision, 0.79 (95% CI, 0.72–0.85) for radiologic feature recognition, and 0.84 (95% CI, 0.78–0.89) for management logic, indicating good to excellent agreement.

Secondary evaluation measures included physician confidence, decision time, and clinician-perceived usability. Confidence was recorded as a percentage, decision time was recorded in seconds. Clinician-perceived usability was recorded on a 1–5 rating scale after physicians reviewed the standardized system output format, and was intended to capture perceived acceptability and clarity of the output presentation rather than usability in a real-time clinical workflow. These endpoints were specified to support analyses of diagnostic performance, physician-rated clinical evaluation, decision dynamics, and clinician-perceived usability in the Results section.

### 2.8 Statistical analysis

Binary diagnostic accuracy was calculated as the proportion of correctly classified observations within the relevant analysis unit. System accuracy used cases as observations, whereas physician accuracy used physician-case evaluation records as observations. For a set of N observations, accuracy was defined as shown in Equation 4:


$$\begin{array}{c} \text{Accuracy}=\frac{1}{N}\sum_{i=1}^{N}I({\hat{y}}_{i}=y_{i}) \end{array}$$ (4)

where N denotes the number of observations in the relevant analysis unit, ${\hat{y}}_{i}$ denotes the predicted or submitted diagnosis for observation i , $y_{i}$ denotes the corresponding validation diagnosis label, and I(·) denotes the indicator function, equal to 1 when the expression is true and 0 otherwise. Accuracy estimates were summarized overall and by evaluator group and case-difficulty stratum. For proportions reported in Table 2, 95% confidence intervals were presented with the corresponding accuracy estimates.

**Table 2:** Diagnostic performance by evaluator group and case difficulty.

Metric	Hip-Agent	Consultants	Residents/fellows	Kappa	*p* value
Overall accuracy (%)	85.1% [76.8–93.4%]	94.6% [92.3–96.9%]	73.5% [70.3–76.7%]	0.82	0.029
Accuracy–easy cases	94.4% [86.6–100.0%]	97.8% [95.6–100.0%]	80.6% [76.4–84.7%]	0.89	0.039
Accuracy–moderate cases	92.0% [80.6–100.0%]	95.2% [91.4–99.0%]	67.6% [61.8–73.4%]	0.84	0.011
Accuracy–complex cases	46.2% [14.8–77.5%]	84.6% [75.6–93.6%]	65.4% [57.1–73.7%]	0.58	0.172

Values are accuracy [95% CI]. *p* values compare the Hip-Agent with residents/fellows. CI, confidence interval.

Rating-domain and secondary continuous measures were summarized using means and standard deviations. For evaluator group g and rating domain m , the group-level mean rating was defined as shown in Equation 5:


$$\begin{array}{c} {\overset{ˉ}{r}}_{g,m}=\frac{1}{n_{g}}\sum_{i=1}^{n_{g}}r_{i,m} \end{array}$$ (5)

where g denotes the evaluator group, m denotes the rating domain, $n_{g}$ denotes the number of observations in group g , and $r_{i,m}$ denotes the rating for observation i in domain m . This aggregation was applied to diagnostic precision, radiologic feature recognition, management logic, physician confidence, decision time, and clinician-perceived usability as appropriate for each measure, with summarized values reported in Table 3.

Inter-observer reliability for the three 5-point clinical-domain ratings was assessed using a two-way random-effects intraclass correlation coefficient (ICC) model for absolute agreement, reported with 95% confidence intervals (25). Kappa values were treated as agreement statistics for diagnostic performance summaries (25). *p* values were reported only for comparisons specified in the statistical summary, and statistical interpretation was limited to those comparisons. Secondary visualizations, including Bland–Altman agreement plots where applicable, were used to characterize distributional patterns in score alignment, confidence, decision time, error rates, and usability ratings, without drawing causal inferences regarding physician behavior or patient outcomes (26).

## 3 Results

### 3.1 Validation set and evaluator composition

The retrospective validation comprised 74 hip-joint cases evaluated by 15 physicians, yielding 1,110 physician-case evaluations (Table 1). The evaluator panel included 5 consultants and 10 residents/fellows, providing two physician seniority groups for comparison across the validation set. This structure allowed diagnostic and clinical rating outcomes to be summarized at both the case level and the physician-case evaluation level.

The case set covered five hip-joint disease categories: osteoarthritis (26 cases), developmental dysplasia of the hip (22 cases), avascular necrosis of the femoral head (15 cases), fracture (8 cases), and labral tear (3 cases). Difficulty strata included 36 easy cases, 25 moderate cases, and 13 complex cases. These disease-category and difficulty-stratum distributions defined the clinical scope of the retrospective evaluation.

### 3.2 Diagnostic performance in the retrospective validation set

**Figure 3:**
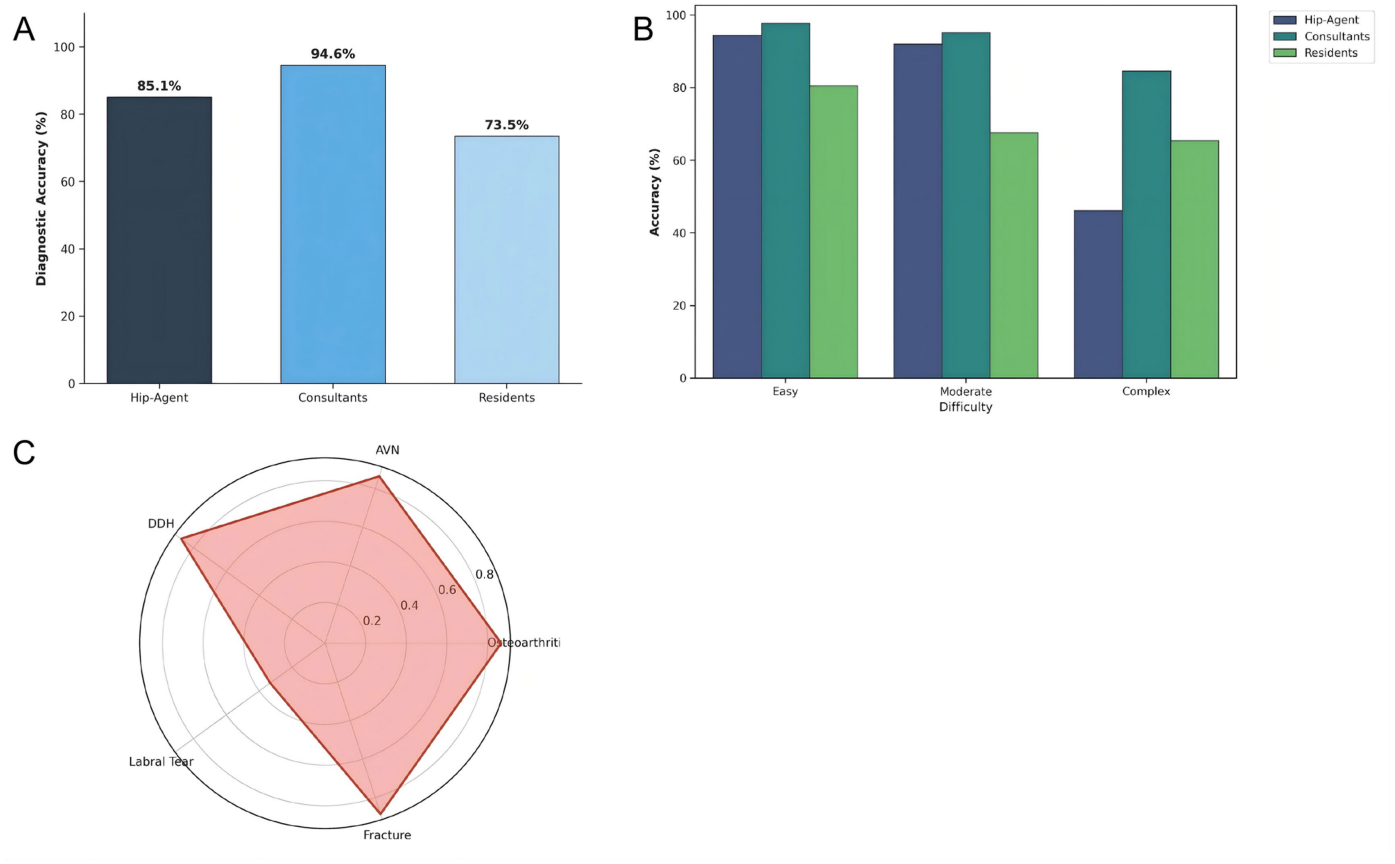
Diagnostic performance across clinical strata. **(A)** Overall binary diagnostic accuracy is shown for the Hip-Agent, consultants, and residents/fellows. **(B)** Accuracy is stratified by case difficulty. **(C)** Hip-Agent accuracy is shown by disease category.

Overall binary diagnostic performance is shown in Figure 3A and summarized in Table 2. Consultants retained the highest overall accuracy at 94.6% (95% CI, 92.3–96.9), while residents/fellows achieved 73.5% (95% CI, 70.3–76.7). The Hip-Agent achieved an overall accuracy of 85.1% (95% CI, 76.8–93.4), placing its performance numerically between the consultant and resident/fellow groups in this retrospective validation set. It should be noted that the validation diagnosis labels were established by an independent expert panel using clinical records and follow-up data; however, because the final diagnoses in the medical record may partly reflect specialist assessment, some degree of incorporation bias cannot be entirely excluded, and the consultant accuracy estimate should be interpreted with this consideration in mind.

Agreement and group-comparison statistics were consistent with this overall pattern. The *κ* statistic for the overall diagnostic comparison was 0.82. The comparison between the Hip-Agent and residents/fellows yielded *p* = 0.029, as summarized in Table 2.

### 3.3 Case-complexity and disease-category analyses

Difficulty-stratified performance showed a clear gradient across case complexity (Figure 3B and Table 2). Hip-Agent accuracy was higher in easy and moderate cases and lower in complex cases, indicating that complex cases represented the most challenging subset for the system in this retrospective validation set. Consultant accuracy also declined in complex cases, although it remained higher than resident/fellow accuracy across difficulty strata.

In easy cases, accuracy was 94.4% (95% CI, 86.6–100.0) for the Hip-Agent, 97.8% (95% CI, 95.6–100.0) for consultants, and 80.6% (95% CI, 76.4–84.7) for residents/fellows; the *κ* statistic was 0.89, and the comparison between the Hip-Agent and residents/fellows yielded *p* = 0.039. In moderate cases, accuracy was 92.0% (95% CI, 80.6–100.0) for the Hip-Agent, 95.2% (95% CI, 91.4–99.0) for consultants, and 67.6% (95% CI, 61.8–73.4) for residents/fellows; the *κ* statistic was 0.84, and the comparison between the Hip-Agent and residents/fellows yielded *p* = 0.011.

In complex cases, accuracy was 46.2% (95% CI, 14.8–77.5) for the Hip-Agent, 84.6% (95% CI, 75.6–93.6) for consultants, and 65.4% (95% CI, 57.1–73.7) for residents/fellows; the κ statistic was 0.58, and the comparison between the Hip-Agent and residents/fellows yielded *p* = 0.172. The disease-category plot suggested visually higher Hip-Agent accuracy for osteoarthritis, avascular necrosis of the femoral head, developmental dysplasia of the hip, and fracture, with lower plotted accuracy for labral tear (Figure 3C). The labral tear category represented a small subgroup of 3 cases in the validation set (Table 1).

### 3.4 Physician ratings of hip-agent output quality

**Figure 4:**
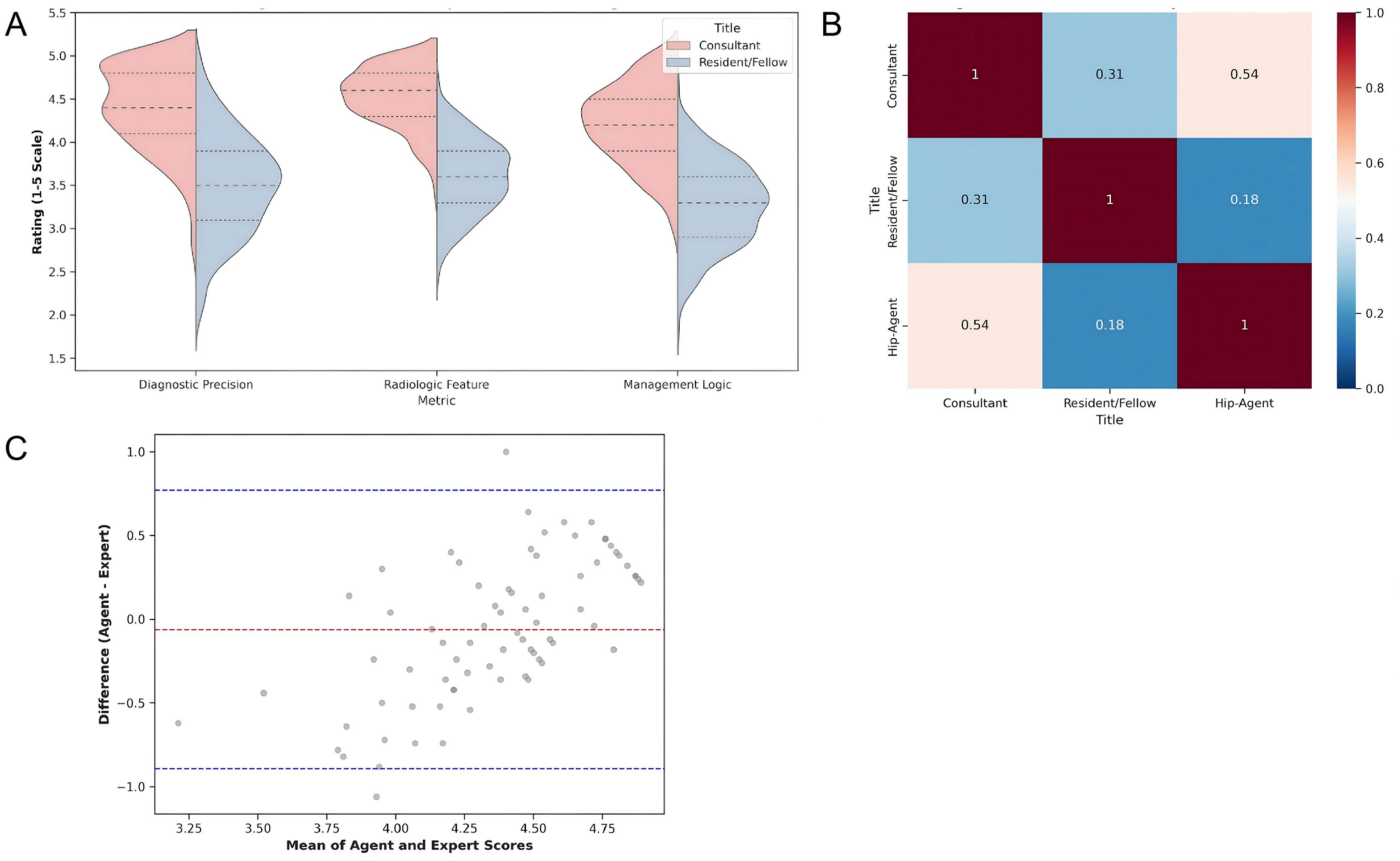
Physician ratings of hip-agent output quality. **(A)** Evaluator ratings of de-identified hip-agent output quality are shown by physician seniority group across diagnostic precision, radiologic feature recognition, and management logic. **(B)** Pearson correlations are displayed for exploratory assessment of rating patterns. **(C)** A Bland–Altman plot displays score differences across the observed scoring range.

Physician ratings of Hip-Agent output quality are shown in Figure 4A and summarized in Table 3. The three domains captured complementary dimensions of the system’s structured output: diagnostic precision, radiologic feature recognition, and management logic. Across all three domains, consultant ratings were numerically higher than resident/fellow ratings, consistent with more experienced evaluators applying more stringent quality criteria to the system outputs.

**Table 3:** Physician-rated hip-agent output quality and secondary evaluation measures.

Measure	Unit	Consultants	Residents/fellows	Overall
Diagnostic precision	1–5 score	4.39 (0.49)	3.51 (0.59)	3.80 (0.69)
Radiologic feature recognition	1–5 score	4.52 (0.34)	3.61 (0.42)	3.91 (0.59)
Management logic	1–5 score	4.20 (0.46)	3.28 (0.48)	3.59 (0.64)
Physician confidence	%	92.0 (4.3)	74.8 (8.7)	80.5 (11.1)
Decision time	Seconds	212.7 (101.9)	212.1 (102.4)	212.3 (102.2)
Usability rating	1–5 score	4.60 (0.49)	4.60 (0.49)	4.60 (0.49)

Values are mean (SD). Diagnostic precision, radiologic feature recognition, and management logic refer to evaluator ratings of de-identified Hip-Agent output quality, not independent physician diagnostic performance. Usability was scored on a 1–5 scale as perceived acceptability of the output format.

For diagnostic precision, the mean score was 4.39 (0.49) among consultants, 3.51 (0.59) among residents/fellows, and 3.80 (0.69) overall. For radiologic feature recognition, the corresponding scores were 4.52 (0.34), 3.61 (0.42), and 3.91 (0.59). For management logic, scores were 4.20 (0.46) among consultants, 3.28 (0.48) among residents/fellows, and 3.59 (0.64) overall. Correlation-based and Bland–Altman visualizations provided complementary descriptive views of score alignment and score dispersion across the observed scoring range (Figures 4B,C).

### 3.5 Decision dynamics and secondary evaluation measures

**Figure 5:**
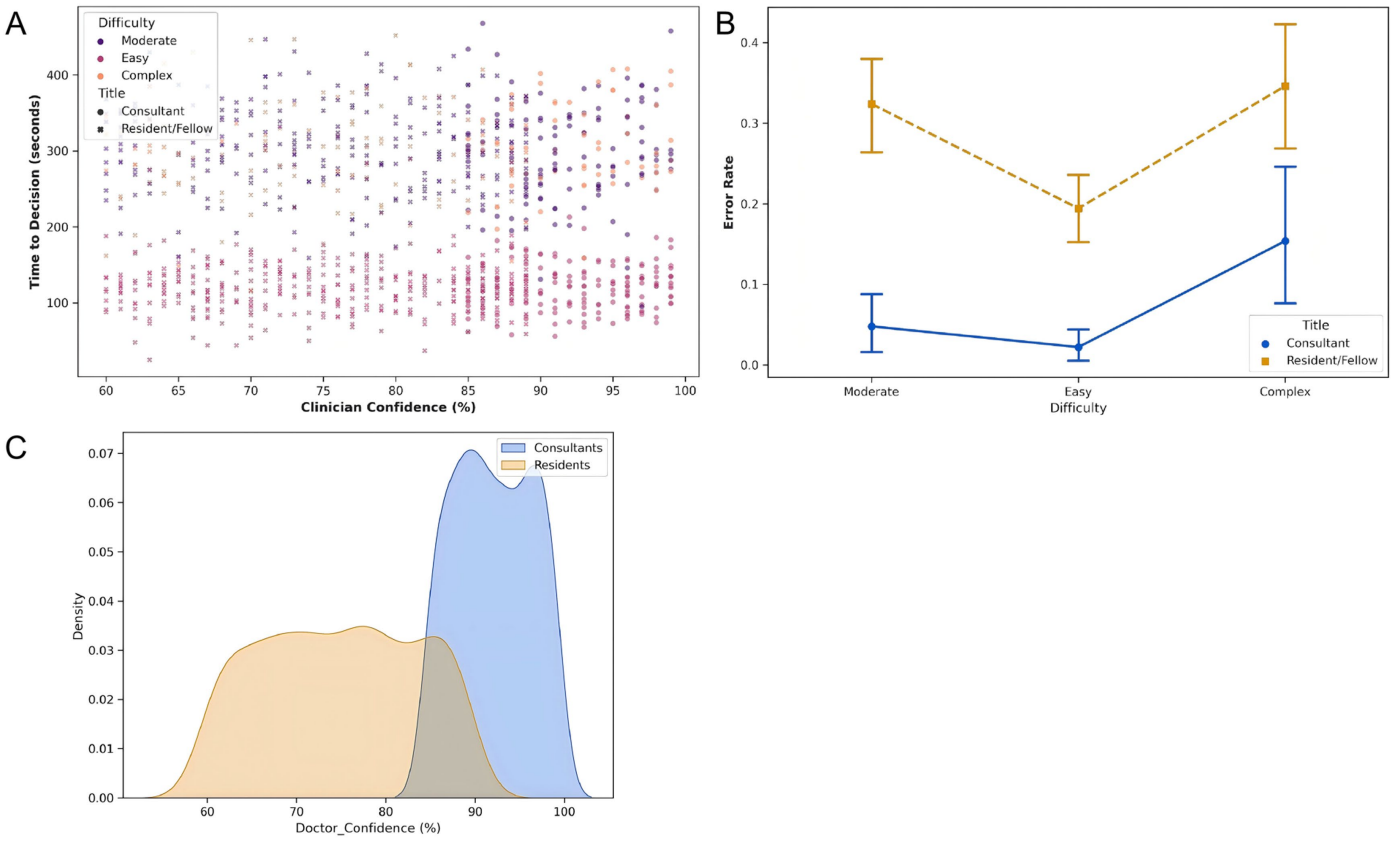
Decision dynamics and secondary evaluation measures. **(A)** Physician confidence and decision time are displayed by case difficulty and physician seniority**. (B)** Diagnostic error rates are summarized by case difficulty and physician seniority. **(C)** Confidence distributions are shown for consultants and residents/fellows.

Decision dynamics and secondary evaluation measures are shown in Figures 5A–C and summarized in Table 3. Physician confidence differed by seniority group, with consultants reporting a mean confidence of 92.0 (4.3)% and residents/fellows reporting 74.8 (8.7)%. The overall mean confidence was 80.5 (11.1)%.

Mean decision time was nearly identical between physician groups: 212.7 (101.9) seconds among consultants and 212.1 (102.4) seconds among residents/fellows, with an overall mean of 212.3 (102.2) seconds. Figure 5A provides a distributional view of the relationship between decision time and confidence across physician seniority and case-difficulty strata. Figure 5B summarizes diagnostic error rates across physician seniority groups and difficulty strata, and Figure 5C displays the confidence-density pattern by physician seniority. Together, these secondary measures characterize physician evaluation patterns within the retrospective validation dataset.

### 3.6 Usability rating distribution

**Figure 6:**
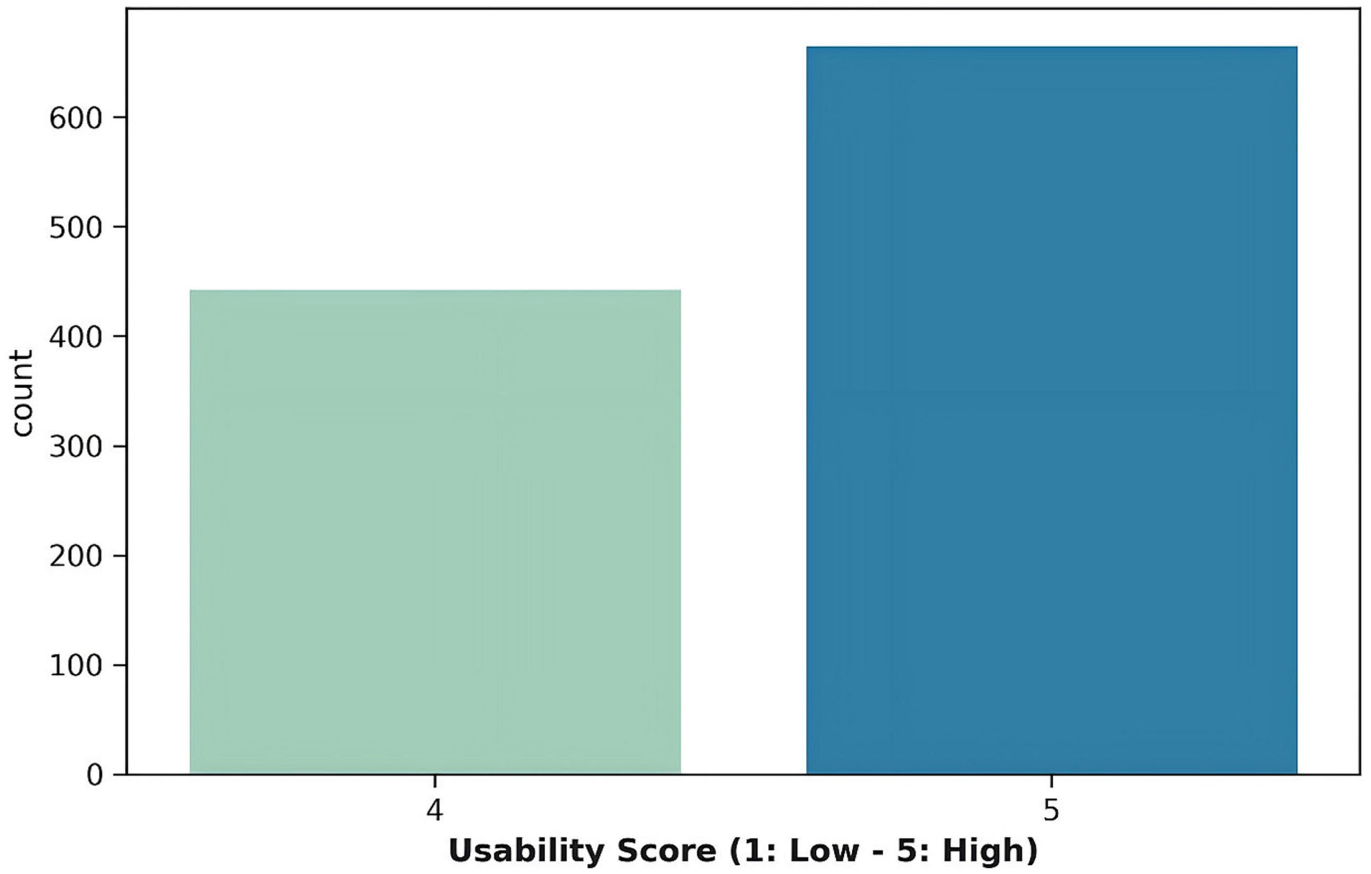
Single-item usability rating distribution. The distribution of 1–5 usability ratings is shown across physician-case evaluations.

Clinician-perceived usability is shown in Figure 6 and summarized in Table 3. On the 1–5 rating scale, usability ratings were 4.60 (0.49) among consultants, 4.60 (0.49) among residents/fellows, and 4.60 (0.49) overall. The distribution was concentrated toward favorable ratings, and the mean usability rating was consistent across physician seniority groups in the retrospective evaluation setting. These ratings reflect perceived acceptability and clarity of the output presentation format rather than usability in a real-time clinical workflow.

## 4 Discussion

This retrospective validation provides an initial assessment of a clinician-facing retrieval-augmented decision-support system for hip-joint disease evaluation. We emphasize that the present study constitutes a retrospective, case-based comparison of the standalone diagnostic performance of Hip-Agent with that of consultant and resident/fellow physician groups, rather than a prospective evaluation of the system’s decision-support effect in an interactive clinical workflow. The Hip-Agent showed intermediate overall diagnostic performance between consultants and residents/fellows, while consultants retained the highest accuracy. Performance was higher in easy and moderate cases and lower in complex cases, indicating that case complexity remained an important determinant of diagnostic performance. Physician-rated domains further showed numerical differences by seniority group across diagnostic precision, radiologic feature recognition, and management logic. Confidence varied by physician seniority, decision time was similar between groups, and perceived acceptability of the output format was high within the retrospective review setting.

The clinical relevance of these findings lies in the heterogeneous nature of hip-joint assessment. Hip pain and functional limitation can reflect degenerative, ischemic, developmental, traumatic, or intra-articular pathology, and these entities often require synthesis of symptoms, radiographs, magnetic resonance imaging, staging or classification systems, and management context (1–8). A retrieval-grounded support system is therefore most appropriately interpreted as a tool for organizing clinical information and disease-specific knowledge rather than as a stand-alone diagnostic authority. In this study, the system was designed to connect case input with relevant hip-joint knowledge and to return structured outputs covering diagnosis, staging or classification support, treatment-support recommendations, and further examination suggestions. Such organization may be useful in clinical domains where the diagnostic label, severity assessment, and management pathway are closely linked.

The difficulty-stratified findings also provide clinically important context. Easy and moderate cases may contain more recognizable combinations of symptoms and imaging features, allowing retrieved disease knowledge and structured prompts to align more readily with the expected diagnosis. Complex cases are more likely to involve overlapping clinical or imaging features suggesting multiple differential diagnoses, atypical presentation, incomplete or subtle imaging features, or management-relevant ambiguity. These factors are also encountered in routine hip assessment, particularly when osteoarthritis, osteonecrosis, dysplasia-related degeneration, fracture, and intra-articular pathology overlap clinically or radiographically (3–6). The lower Hip-Agent accuracy in complex cases should therefore be interpreted as a signal that more difficult case phenotypes require further refinement, broader representation during development, and careful physician oversight. Importantly, incorrect AI-generated suggestions in complex cases may pose risks to clinical judgment, including automation bias—whereby clinicians may unduly trust system outputs—and the potential for erroneous recommendations to misdirect diagnostic reasoning or delay appropriate management. These considerations underscore the need for caution when deploying such systems in diagnostically challenging scenarios and suggest that, until performance in complex cases improves, the system should be used primarily as an adjunctive information source with explicit physician verification rather than as a primary decision tool for complex presentations. The labral tear subgroup also requires cautious interpretation because it represented only a small number of cases in this validation set.

The comparison across physician groups supports a clinician-facing interpretation of the system. The Hip-Agent performance pattern was numerically between consultants and residents/fellows overall, while consultant performance remained highest. This pattern is consistent with a role in structured clinical reasoning rather than replacement of senior clinical judgement. For residents and fellows, tools that make diagnostic reasoning more explicit may help standardize attention to key symptoms, imaging signs, and classification cues; for experienced clinicians, the same outputs may serve as a structured second-pass summary or evidence-linked checklist. These proposed roles remain hypotheses generated from the present retrospective findings rather than demonstrated clinical effects. Such use is aligned with broader principles for clinical decision support, which emphasize human oversight, interpretability, workflow fit, and the clinician’s continuing responsibility for the final decision (9, 14, 17). The present retrospective design supports a plausible role for structured decision support, which should be examined in future studies that explicitly evaluate physician interaction with system outputs.

The system architecture also illustrates how retrieval-augmented and task-decomposed workflows may be adapted for specialty decision support. Medical large language models can organize clinical text, but their outputs require grounding, evaluation, and governance because unsupported generation remains a recognized concern (27–32). Retrieval-augmented generation may support traceability by conditioning generation on selected knowledge segments, and healthcare RAG reviews emphasize both the promise of evidence grounding and the need for careful source curation and evaluation (22–24). In the present system, hybrid retrieval combined semantic and lexical matching, including BM25-based sparse retrieval, to improve access to both conceptually similar passages and exact disease or staging terminology (21). The multi-agent or task-decomposed workflow further organized outputs into clinically meaningful components, including diagnosis, classification or staging support, treatment recommendation, and further examination suggestion. This structure may help reduce unstructured narrative variability, although it does not remove the need for clinical review.

Within this context, the favorable perceived acceptability of the output format within the retrospective review setting supports further evaluation of output clarity and presentation, and provides a basis for subsequent prospective workflow evaluation in which real-time usability can be properly assessed.

Several considerations should be noted when interpreting these findings. This was a retrospective, single-center validation study, and performance may vary with institutional practice, imaging protocols, physician experience, and case-mix composition. The sample size was modest, with limited representation of some disease categories, particularly labral tear, so subgroup-level findings should be interpreted as exploratory. The current validation dataset focused on case-level diagnostic and evaluation endpoints and did not incorporate complete demographic, staging, or longitudinal follow-up information; complete standardized staging information was not available for all retrospective cases, and disease severity distributions are provided as descriptive summaries (Supplementary Table S2). A key limitation is that the study design did not incorporate a human-AI interaction component: we did not assess whether clinicians’ diagnostic accuracy, confidence, decision time, or error rates changed when using the system, nor did we evaluate patterns of acceptance, modification, or rejection of AI suggestions. Consequently, the results should not be interpreted as evidence of a clinical decision-support effect in an active workflow. The clinical-domain rating scales were study-specific descriptive measures rather than validated psychometric instruments. Additionally, although the validation diagnosis labels were established by an independent expert panel, the possibility of incorporation bias cannot be entirely excluded, as the final diagnoses in medical records may partly reflect the assessments of specialists. Future studies should use prospectively defined interaction workflows, more detailed reference-standard adjudication, prespecified difficulty assignment, and broader multi-institutional case sets to evaluate generalizability and clinical integration. These considerations are consistent with early-stage AI evaluation and support a measured interpretation of retrospective feasibility (17, 33).

Future work should evaluate the system in prospective clinical workflows, with predefined physician interaction points, external multicenter cohorts, and richer representation of complex and underrepresented disease categories. Additional development should focus on complex-case handling, subgroup calibration, retrieval-quality monitoring, and explicit evaluation of how physicians interpret and act on system outputs. Before clinical implementation, decision-support systems require ongoing monitoring for generalizability, workflow integration, model drift, and user behavior across local care pathways (34–36). Overall, this study supports the feasibility of retrieval-augmented, structured decision support for retrospective hip-joint case evaluation and provides a foundation for future prospective workflow evaluation in broader clinical settings.

## 5 Conclusion

This retrospective case-based validation study evaluated a clinician-facing decision-support system for hip-joint disease assessment that integrated a local medical knowledge base, retrieval-augmented generation, and a structured multi-agent reasoning workflow. The system demonstrated feasible retrospective performance as a decision-support tool, with overall diagnostic accuracy positioned between consultant and resident/fellow physician groups. Performance varied by case complexity, with stronger results in easy and moderate cases and lower accuracy in complex cases, emphasizing the continued importance of physician oversight in diagnostically challenging presentations. Physician-rated domains and usability findings further suggest that structured outputs were interpretable within the retrospective evaluation workflow. Because physicians did not use the system to revise their decisions, the present findings should not be interpreted as evidence that Hip-Agent improves clinician performance in practice. Future studies should examine prospective workflow use, broader multi-institutional case sets, underrepresented disease categories, and human-AI interaction to clarify generalizability and clinical integration.

## Data Availability

The data analyzed in this study is subject to the following licenses/restrictions: the data analyzed in this retrospective study are not publicly available due to privacy and confidentiality restrictions, as they contain sensitive patient information from a single institution. De-identified data may be obtained from the corresponding author upon reasonable request, subject to approval by the institutional ethics committee (Second Affiliated Hospital of Guizhou Medical University) and compliance with applicable data protection regulations. Requests to access these datasets should be directed to Q-YL, sdlqy123@163.com.
