# Supplementary material for: Retrieval-augmented clinical decision support for structured hip-joint disease assessment
Source: Front Med (Lausanne). 2026 Jul 17;13:1890014. doi: 10.3389/fmed.2026.1890014 (PMC13424293; doi:10.3389/fmed.2026.1890014)
Supplement: Supplementary file 1 [file Supplementary_file_1.docx]

### Supplementary Figure


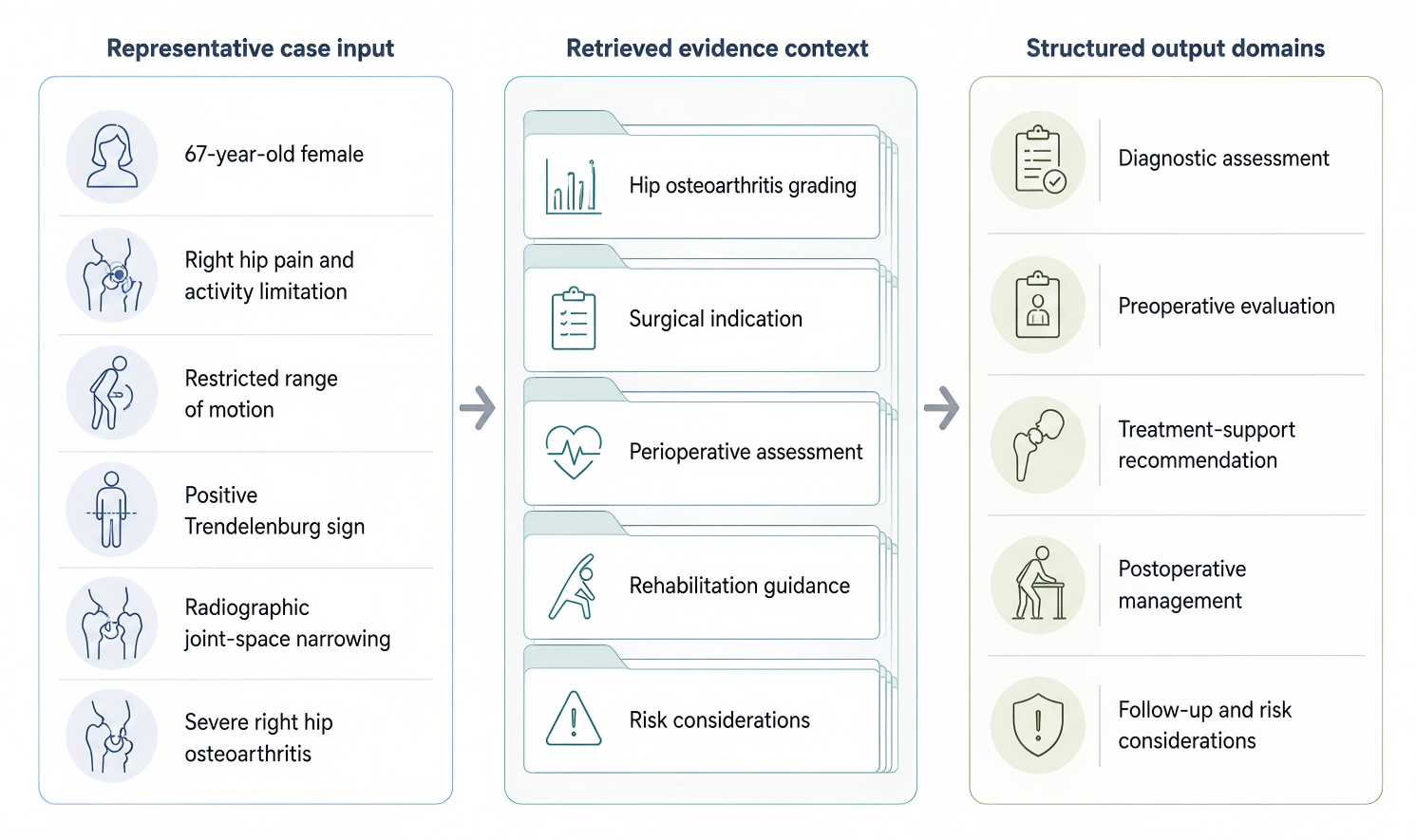


**Supplementary Figure 1. Representative structured output domains for a de-identified hip-joint case vignette.** The illustrative example shows how representative case input and retrieved evidence context were organized into structured output domains, including diagnostic assessment, preoperative evaluation, treatment-support recommendation, postoperative management, and follow-up or risk considerations. This figure illustrates the output format and was not used as comparative validation evidence.

### Supplementary Table

**Supplementary Table S1. Knowledge base source composition**

| **Source Type** | **Count** | **Representative Examples** |
| --- | --- | --- |
| Clinical guidelines | 47 | AAOS osteoarthritis management; AAOS hip fracture management; ACR Appropriateness Criteria for chronic hip pain |
| Expert consensus documents | 23 | ARCO staging system for osteonecrosis; Warwick Agreement on femoroacetabular impingement; DDH management consensus statements |
| Peer-reviewed articles | 156 | Diagnostic criteria, imaging protocols, staging systems, treatment outcomes for the five target diseases |
| Textbook-derived chapters | 8 | Disease knowledge chapters covering OA, DDH, AVN, fracture, and labral pathology |
| Terminology resources | 5 | Synonym normalization and cross-referencing databases |

Note. Source selection was performed by two orthopedic consultants based on clinical relevance, methodological quality, and temporal currency (sources published within the preceding 10 years prioritized, with landmark older references retained). Inclusion required agreement between both selectors, with discrepancies resolved by a third senior consultant. The indexed knowledge base contained 2,847 discrete knowledge segments after segmentation and cleaning.

**Supplementary Table S2. Disease severity and classification distribution**

| **Disease Category** | **N** | **Severity/Classification Distribution** |
| --- | --- | --- |
| Osteoarthritis | 26 | KL grade 2: 10; grade 3: 9; grade 4: 7 |
| Avascular necrosis | 15 | ARCO stage II: 4; stage III: 6; stage IV: 5 |
| Developmental dysplasia | 22 | Tönnis grade 1: 8; grade 2: 9; grade 3: 5 |
| Fracture | 8 | Femoral neck (Garden II–III): 3; intertrochanteric: 3; subtrochanteric: 2 |
| Labral tear | 3 | MRI-confirmed: 3 |

Note. Complete standardized staging information was not available for all retrospective cases; therefore, disease severity distributions should be interpreted as descriptive summaries of the available data. KL = Kellgren-Lawrence; ARCO = Association Research Circulation Osseous; DDH = developmental dysplasia of the hip.
